# Differential modulation of innate immunity *in vitro* by probiotic strains of *Lactobacillus gasseri*

**DOI:** 10.1186/1471-2180-13-298

**Published:** 2013-12-23

**Authors:** Diomira Luongo, Junki Miyamoto, Paolo Bergamo, Filomena Nazzaro, Federico Baruzzi, Toshihiro Sashihara, Soichi Tanabe, Mauro Rossi

**Affiliations:** 1 Institute of Food Sciences, NRC, Avellino, Italy; 2 Department of Biofunctional Science and Technology, Graduate School of Biosphere Science, Hiroshima University, Higashi, Hiroshima, Japan; 3 Institute of Sciences of Food Production, NRC, Bari, Italy; 4 Division of Research and Development, Meiji Co., Ltd., Odawara, Japan

**Keywords:** *L. gasseri* OLL2809, *L. gasseri* L13-Ia, Mouse dendritic cells, MODE-K cells, Immunomodulation

## Abstract

**Background:**

Probiotics species appear to differentially regulate the intestinal immune response. Moreover, we have shown that different immune-modulatory abilities can be found among probiotic strains belonging to the same species. In this study, we further addressed this issue while studying *L. gasseri*, a species that induces relevant immune activities in human patients.

**Results:**

We determined the ability of two strains of *L. gasseri*, OLL2809 and L13-Ia, to alter cell surface antigen expression, cytokine production and nuclear erythroid 2-related factor 2 (Nrf2)-mediated cytoprotection in murine bone marrow-derived dendritic cells (DCs) and MODE-K cells, which represent an enterocyte model. Differential effects of *L. gasseri* strains were observed on the expression of surface markers in mature DCs; nevertheless, both strains dramatically induced production of IL-12, TNF-α and IL-10. Distinctive responses to OLL2809 and L13-Ia were also shown in MODE-K cells by analyzing the expression of MHC II molecules and the secretion of IL-6; however, both *L. gasseri* strains raised intracellular glutathione. Treatment of immature DCs with culture medium from MODE-K monolayers improved cytoprotection and modified the process of DC maturation by down-regulating the expression of co-stimulatory markers and by altering the cytokine profile. Notably, bacteria-conditioned MODE-K cell medium suppressed the expression of the examined cytokines, whereas cytoprotective defenses were significantly enhanced only in DCs exposed to OLL2809-conditioned medium. These effects were essentially mediated by secreted bacterial metabolites.

**Conclusions:**

We have demonstrated that *L. gasseri* strains possess distinctive abilities to modulate *in vitro* DCs and enterocytes. In particular, our results highlight the potential of metabolites secreted by *L. gasseri* to influence enterocyte-DC crosstalk. Regulation of cellular mechanisms of innate immunity by selected probiotic strains may contribute to the beneficial effects of these bacteria in gut homeostasis.

## Background

The intestinal microbiota interacts with the local immune system to promote mechanisms of intestinal homeostasis and health. Many studies have provided evidence that probiotics can also effectively modulate the gut immune system in health and disease [1]. In particular, probiotic bacteria influence both the development and regulation of intestinal immune responses and non-immune defenses [2]. The symbiosis between human hosts and gut microbes has risks and benefits for the host organism as bacteria continuously challenge intestinal immune homeostasis with microbial-associated molecular patterns (MAMPs). However, the risks of an exaggerated inflammatory response and chronic inflammation are limited by the polarized expression of pattern recognition receptors intracellularly or on the basolateral membrane of epithelial cells (ECs) and dendritic cells (DCs) that intercalate between ECs for direct bacterial uptake [3].

Paradoxically, little information is available regarding probiotics that possess physiologically relevant anti-oxidant properties. Nevertheless, a large body of evidence confirms that high-grade oxidative stress is one of the crucial players in the pathogenesis of disorders such as inflammatory diseases. Accumulating data suggest that the nuclear erythroid 2 p45-related factor 2 (Nrf2) is a key regulatory transcription factor that induces defense-related genes that protect against the deleterious effects of reactive oxygen species (ROS) and that targeted activation of this transcription factor could represent a therapeutic approach for the treatment of inflammatory diseases [4]. Nrf2 is a redox-sensitive, basic leucine zipper transcription factor. During oxidative stress, following detachment from a cytosolic inhibitor (Keap1), Nrf2 translocates to the nucleus and leads to transcription of antioxidant and detoxifying enzymes involved in detoxification and chemoprevention such as glutathione S-transferases (GSTs), NAD(P)H:quinone oxidoreductase (NQO1) and gamma-glutamylcysteine ligase (γGCL), which is the rate-limiting enzyme for glutathione (GSH) synthesis [5]. Bacteria-induced ROS generation greatly influences eukaryotic signaling pathways including those inducing Nrf2 [6,7], and improved Nrf2-mediated protection is associated with beneficial effects elicited by probiotic intake [8,9].

When studying host responses, there is a tendency to focus on individual cell types that comprise the biological barriers to microorganisms to obtain information on a particular cellular reaction to a microbe. Specifically, *in vitro* studies have focused on interactions between probiotics and enterocytes. The immunomodulatory role of the intestinal epithelium is attracting considerable attention, in addition to its well-known role in barrier function. In analyses of enterocytes, it was shown that *Bifidobacterium infantis* and *Lactobacillus salivarius* did not induce proinflammatory responses in human intestinal epithelial cells (IECs) compared with the responses generated by *Salmonella typhimurium*, suggesting that IECs display immunological unresponsiveness when exposed to LAB [10]. Using a co-culture model including Caco-2 (IEC) and PBMC cells, Haller et al. also observed differential IEC activations between *Escherichia coli* and LAB strains [11]. Furthermore, Rimoldi et al. reported that the release of pro-inflammatory mediators by IECs in response to bacteria is dependent on bacterial invasiveness and the presence of flagella in a human co-culture system [12]. Other relevant studies have focused on dendritic cells (DCs), canonical antigen-presenting cells, that can effectively induce primary immune responses against microbial infections and other stimuli [13,14]. A recent report demonstrated that individual strains from the *Lactobacillus* group can differentially regulate the expression of surface markers and cytokine production by DCs [15]. By using human DCs as a model, it was shown that bacterial strains belonging to different species display distinct immunomodulatory effects [16]. Moreover, different strains of the same species can also differentially polarize the immune response [17,18]. Recently, we have examined this aspect by focusing on *L. paracasei* that we have found to induce the highest maturation degree of DCs among the tested species [19]. In particular, we observed a differential ability of five genetically characterized *L. paracasei* strains to modulate DCs [20]. In this study, we addressed the same question by studying *L. gasseri*. We focused on *L. gasseri* because this species induces relevant immune activities in human patients [21]. To simulate the interactions occurring in the intestinal mucosa, we challenged *in vitro* MODE-K cells, a mouse model of enterocyte, and murine bone marrow DCs with two *L. gasseri* strains. Our work indicated the existence of strain-specific effects of *L. gasseri*. This modulatory activity was found to be associated with the production of bacterial metabolites distinctively impacting both the immune and anti-oxidant properties of IECs and DCs.

## Methods

### Bacterial strains and culture conditions

*Lactobacillus gasseri* OLLL2809 (from human intestine; deposited in the Patent Microorganisms Depositary, National Institute of Technology and Evaluation, Japan, Accession n. NITE BP-72) and L13-Ia (from raw bovine milk, deposited in the Microbial Culture Collection, Institute of Sciences of Food Production, Italy, Accession n. 13541) were studied. Strain OLL2809 is considered to be a probiotic strain [22], while potential probiotic features of strain L13-Ia, able to resist to simulated gastric and pancreatic digestion, as well as to bovine and porcine bile salts were previously demonstrated [23]. Working cultures were grown in deMan Rogosa Sharpe (MRS) broth (Difco, Detroit, Michigan, USA) for 24 h at 37°C under aerobic conditions without shaking, and these cultures were subcultured twice before use in experiments. The cell concentration of individual strains was evaluated by measuring the optical density at 600 nm and converting this value to the corresponding CFU ml^-1^ value. Before eukaryotic cell challenge, bacterial strains were irradiated with 2800 Gy (Gray) γ-irradiation (MDS Nordion γ-cell 1000) to prevent their proliferation.

### Antimicrobial activity

The antimicrobial activity was assessed by using the inhibition halo test. The pathogenic *Bacillus cereus* (DSM 4313 and DSM 4384), *Escherichia coli* (DSM 8579) and *Pseudomonas aeruginosa* species were used as tester strains. The two strains of *Lactobacillus gasseri* were grown in MRS broth at 37°C to 1 × 10^6^ CFU ml^-1^. Cells were centrifuged at 5000 × g for 15 min at 4°C and collected supernatant was filtered through a 0.22 μm filter before use for the test. Different volumes of supernatants were spotted onto sterile filter disks with a diameter of 5 mm that were plated onto TY (Tryptone Yeast extract, Difco) agar plates previously inoculated with the pathogen tester strains. The TY agar plates were then incubated at 37°C for 24–48 hours. DMSO was used as negative control; gentamycin (8 μg/disc) and tetracycline (7 μg/disc) were used as positive controls. The test was performed in triplicate.

### IEC cell line

MODE-K cells (H-2 k), a murine small intestinal epithelial cell line [24], were kindly provided by Dr. D Kaiserlian (INSERM, Paris, France). These cells were maintained as adherent cells at 37°C in a humidified atmosphere of 5% CO_2_ in air in RPMI medium (Sigma, St. Louis, MO) containing 25 mM HEPES, 1% nonessential amino acids, 0.055% sodium pyruvate, 10% FCS, and 4 mM L-glutamine (complete RPMI medium). Cells were detached before analysis using a solution of 0.25% trypsin in 0.5 mM EDTA (Sigma, St. Louis, MO). In some experiments, MODE-K cells were treated with recombinant murine TNF-α (5 μg l^-1^, PharMingen, San Diego, CA) for 24 h.

### Mice

B10.M mice were maintained under pathogen-free conditions at the animal facility of the Institute of Food Sciences. Mice were used at the age of 6–12 weeks and were euthanized by inhalation of anesthesia with isoflurane. These studies were approved by the National Institutional Review Committee.

### Isolation of bone marrow-derived dendritic cells

Murine DCs were generated according to a previously published method [25]. In brief, bone marrow cells from the femurs and tibiae of mice were flushed and bone marrow cell aliquots (2 × 10^6^) were diluted in 10 ml of RPMI 1640 medium supplemented with 25 mM HEPES, antibiotics (penicillin 100 IU ml^-1^; streptomycin 100 IU ml^-1^), 10% fetal calf serum and 20 ng ml^-1^ granulocyte-macrophage colony-stimulating factor (GM-CSF) (culture medium) before being seeded in 100-mm petri dishes (Falcon, Heidelberg, Germany). On day 3, 10 ml of culture medium was added, and on day 7, 10 ml of the culture medium was replaced with freshly prepared medium. On day 9, non-adherent DCs were harvested by gentle pipetting. Cell aliquots (1 × 10^6^ ml^-1^) were then placed in 24-well plates and incubated in culture medium with 5 ng ml^-1^ GM-CSF in the presence of 1 μg ml^-1^ LPS for 6 h (LPS pulse) to induce the maturation of iDCs. Cell viability was microscopically evaluated by dye-exclusion test using Nigrosin (1% solution) and found ≥ 90% live cells in all experiments.

### Microbial challenge

Confluent epithelial MODE-K cell monolayers or DCs (1 × 10^6^ ml^-1^) were incubated for 24 h with irradiated bacteria resuspended in complete RPMI medium at a 30:1 bacteria: eukaryotic cell ratio. Following incubation, cells were analyzed by Nigrosin and ≥ 90% live cells were still found. Conditioned media were centrifuged at 10000 × g 10 min to eliminate any residual cells and cell debris and supernatants stored at -80°C. No pH change occurred in the medium after 24 h of bacteria incubation. In crosstalk experiments, iDCs were treated with supernatants from the MODE-K cell culture for 24 h, then LPS-pulsed and cultured for additional 24 h in complete RPMI medium.

### FACS analysis

DCs were stained with phycoerythrin (PE)- or fluorescein isothiocyanate (FITC)-conjugated Abs (BioLegend, San Diego, CA, USA) against CD11b, CD11c, CD40 and CD80. MODE-K cells were analyzed for MHC class II expression using a FITC-conjugated goat anti-mouse antibody (BioLegend). Cell staining was analyzed using a CyFlow Space flow cytometer (Partec, Munster, Germany) and FlowJo software (Tree Star Inc., Ashland, OR, USA). For each Ab, an isotype control of the appropriate subclass was used.

### Analysis of cytokine production

Supernatants from DCs cultures were analyzed for IL-12, TNF-α and IL-10 protein levels, whereas MODE-K cell supernatants were analyzed for IL-6 expression by sandwich-type ELISA. First, 100 μl of capture antibody solution (BioLegend) were dispensed into each well of a 96-well plate (Nunc Maxisorb; eBioscience Inc., San Diego, CA) and incubated overnight at 4°C. After the removal of the capture antibody solution, 100 μl of PBS supplemented with 2% BSA (blocking buffer) were added to each well and incubated at room temperature for 2 h. Next, cytokine standards and samples diluted in blocking buffer supplemented with 0.05% Tween-20 were added to the respective wells and incubated overnight at 4°C. At the end of the incubation, after three washings steps with PBS supplemented with 0.05% Tween-20, 100 μl of biotinylated antibody solution were added to the wells and incubated for 2 h at room temperature. After three washing steps, streptavidin–horseradish peroxidase conjugate (1:2000 dilution; Biolegend) were then added to the wells and incubated for 1 h at room temperature. Finally, after washing, 100 μl of 63 mM Na_2_HPO_4_, 29 mM citric acid (pH 6.0) containing 0.66 mg ml^-1^ o-phenylenediamine/HCl and 0.05% hydrogen peroxide were dispensed into each well, and the wells were allowed to develop. The absorbance was read at 415 nm and the cytokine concentrations were calculated using standard curves and expressed as pg ml^-1^.

### Cell viability, redox status and phase 2 enzyme activity

Lactate dehydrogenase (LDH) in spent media was measured [26] to determine the effects of the different treatments on eukaryotic cell viability. Release of total thiols [GSHtot, GSH + glutathione disulfide (GSSG)], GSH and GSSG concentrations in cytosolic extracts were quantified using the 5,5′-dithionitrobenzoic acid (DTNB)-GSSG reductase recycling method [27]. Upon normalization to protein content, intracellular GSH and GSSG were expressed as nmoles mg^-1^ min^-1^. The extracellular thiol level was expressed as nmoles min^-1^. NQO1 and GST activities were measured in cytosolic extracts as previously described [28], and the obtained values were normalized to the protein content and expressed as nmoles 1-chloro-2,4-dinitrobenzene (CDNB) mg^-1^ min^-1^ and nmoles NAD mg^-1^ min^-1^, respectively.

### Statistical analysis

Statistical significance was determined by *t*-test or ANOVA using the GraphPad PRISM 4.0 software (GraphPad Software, Inc., La Jolla, CA). A *P*-value of 0.05 or less was considered to be significant.

## Results

### Probiotic properties of *L. gasseri* OLL2809 and L13-Ia

*L. gasseri* OLL2809 and L13-Ia have been isolated from human intestine and raw bovine milk, respectively, and their properties have previously been reported [22,23]. To further assess these strains’ probiotic features, we focused on their antimicrobial activity. Table 1 shows the inhibition halos produced by L13-Ia and OLL2809 against four pathogenic bacterial strains. The supernatants of both strains were found to be effective against all tested pathogens without significant differences in their inhibitory activity. This indicated that the two strains of *L. gasseri* exhibited similar antimicrobial behavior that, in absence of deeper investigation, could derive by lactic acid production or hydrogen dioxide, both molecules usually released by lactobacilli during their growth. Table 2 summarizes salient characteristics of OLL2809 and L13-Ia.

**Table 1:** **Antimicrobial activity of *Lactobacillus gasseri *** **L13-Ia and OLL2809 as determined by diffusion techniques**

	**Inhibition halo (mm ± SD)**								
**Microorganisms**	**L13-Ia culture supernatant (μl/disc)**			**OLL2809 culture supernatant (μl/disc)**			**DMSO (μl/disc)**	**Gentamycin (μg/disc)**	**Tetracycline (μg/disc)**
	**5**	**10**	**20**	**5**	**10**	**20**	**20**	**8**	**7**
*B. cereus* DSM 4313	4.5 ± 0.5	6.5 ± 0.5	8 ± 0.5	4.5 ± 0.5	6.5 ± 0.15	8 ± 0.35	na	15.3 ± 0.65	9.7 ± 0.7
*B. cereus* DMS 4384	5 ± 0.0	6.5 ± 0.0	7.5 ± 0.0	4.5 ± 0.15	6.5 ± 0.0	8 ± 0.15	na	15.5 ± 0.0	9.65 ± 0.15
*E. coli* DMS 8579	na	3.45 ± 0.45	4.65 ± 0.45	na	3.5 ± 0.4	4.6 ± 0.4	na	15.7 ± 0.4	12.7 ± 0.2
*Ps. aeruginosa*	na	4.65 ± 0.15	7.5 ± 0.4	na	4.65 ± 0.2	7.3 ± 0.2	na	5.7 ± 0.2	4.3 ± 0.15

na, no activity.

**Table 2:** **Key characteristics of *L.gasseri *** **strains used in the study**

**Strain**	**Code**	**Collection**	**Probiotic features**	**References**
OLL2809	16S rRNA partial gene sequence available in GenBank (accession number AB829518).	Meiji Co, Ltd, (Odawara, Japan)	Colonization of human gut; activity in reducing IgE-mediated allergy; growth inhibition of pathogenic species.	[22], this issue
L13-Ia	16S rRNA partial gene sequence available in GenBank (accession number KF934204).	ISPA-CNR (Italy)	Survival to gastric and pancreatic juice treatments; resistance to bile salts; growth inhibition of pathogenic species.	[23], this issue

### Differential effects of *L. gasseri* strains on mature DCs

**Figure 1:**
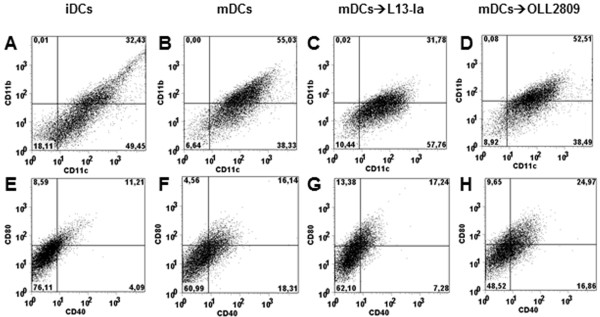
**FACS analysis of BMDCs from B10.M mice.** iDCs were subjected to a 6-h LPS pulse to induce maturation. mDCs were then challenged with irradiated *L. gasseri* OLL2809 or L13-Ia. Both iDCs and treated or untreated mDCs were stained for CD11b and CD11c (panels **A-D**) or CD40 and CD80 (panels **E-H**). Data were collected from ungated cells and are representative of three independent experiments.

**Figure 2:**
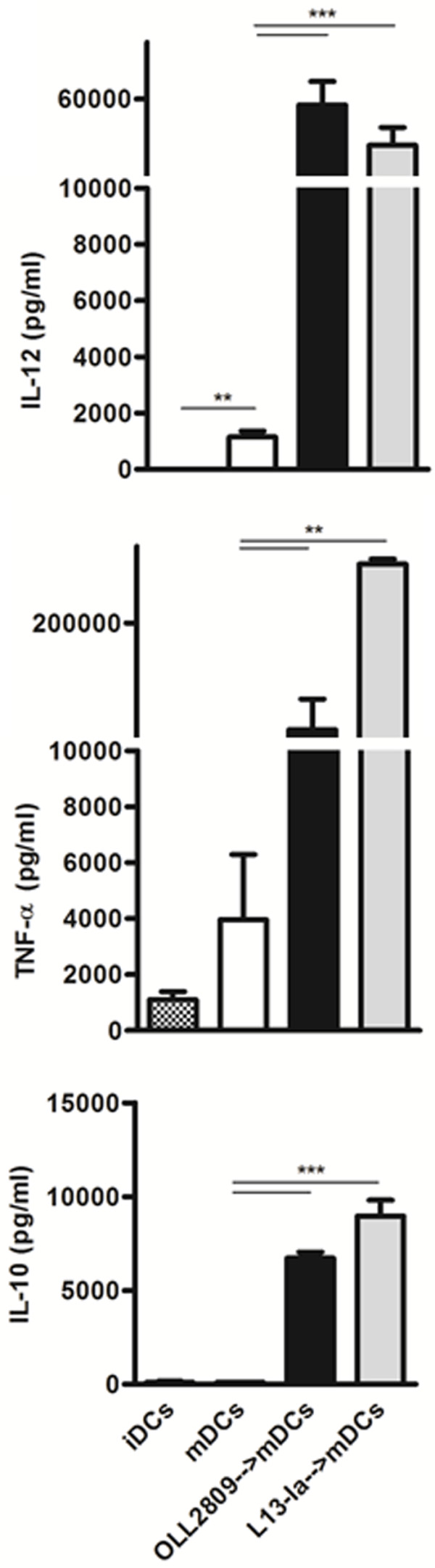
**Cytokine production by mDCs in response to irradiated *L. gasseri *OLL2809 or L13-Ia.** Culture supernatants were collected after 24 h and analyzed for IL-12, TNF-α and IL-10 expression by sandwich-type ELISA; values are expressed in pg/ml; columns represent the mean ± SD and are representative of three independent experiments. **, *P* < 0.01; ***, *P* < 0.001.

Intestinal DCs are able to directly sample luminal antigens by extruding dendrites between epithelial cells [3,29]. To reproduce this interaction *in vitro,* we pulsed bone marrow-derived DCs (≥ 80% CD11c^+^) with LPS to obtain mature DCs (mDCs). Maturation was characterized by an increase in CD11b^+^CD11c^+^DCs (Figure 1A-B). These cells were cultured for 24 h in the presence of irradiated *L. gasseri*. L13-Ia, but not OLL2809, decreased the number of CD11b CD11c double-positive mDCs (32 and 52%, respectively, Figure 1C-D). LPS treatment also caused an increase in the expression of the CD80 and CD40 costimulatory markers (Figure 1E-F). OLL2809, but not L13-Ia, increased the expression of both CD80 and CD40 on mDCs (Figure 1G-H). We next analyzed the effects of irradiated bacteria on the cytokine profile of the DCs. As previously reported [18], LPS induced maturation of DCs derived from this mouse strain and increased the secretion of IL-12 and TNF-α, but not of IL-10 (Figure 2). Notably, *in vitro* challenge with both bacterial strains dramatically enhanced the expression of all examined cytokines including IL-10, showing significant differences with the positive control (mDCs alone; Figure 2).

### Stimulatory activity of *L. gasseri* strains on IECs

**Figure 3:**
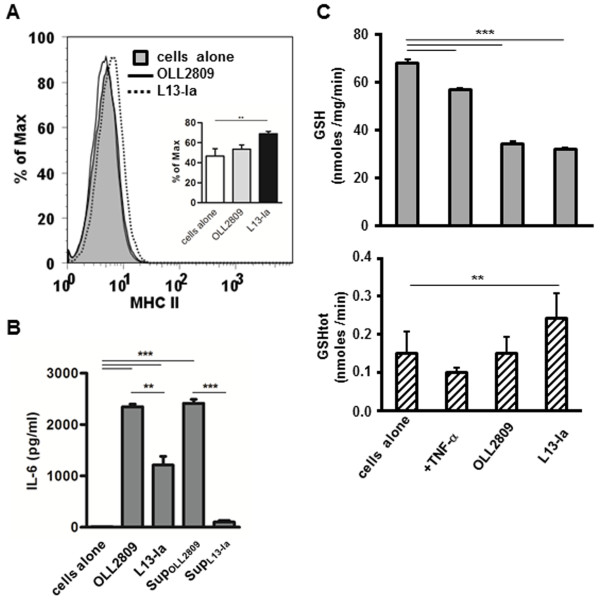
**Effects of *L. gasseri *OLL2809 or L13-Ia on an intestinal cell line. A)** FACS analysis of MHC class II expression in MODE-K cells incubated with irradiated *L. gasseri* OLL2809 or L13-Ia; values are expressed as percentages of the maximal fluorescence intensity. Inset, statistical evaluation of MHC class II expression; columns represent the mean ± SD of three independent experiments; **, *P* < 0.01. **B)** IL-6 production by MODE-K cells following 24 h stimulation with irradiated bacteria or their metabolites (Sup_OLL2809_ and Sup_L13-Ia_); values are expressed in pg/ml. **C)** Intracellular GSH concentration in MODE-K cells, expressed in nmoles/mg prot/min (upper panel), and GSHtot amount in spent media, expressed in nmoles/min (lower panel), following 24 h stimulation with irradiated bacteria; columns represent the mean ± SD and are representative of three independent experiments. sup, supernatant from irradiated bacteria incubated for 24 h in RPMI complete medium. **, *P* < 0.01; ***, *P* < 0.001.

Next, the capacity of OLL2809 and L13-Ia to stimulate enterocytes was investigated. Confluent monolayers of the murine epithelial cell line MODE-K were challenged with irradiated bacteria. IEC viability, evaluated by measuring LDH release in the medium, was not influenced by incubation with bacteria (data not shown). MODE-K cells were then analyzed to determine surface expression of MHC II molecules and secretion of the cytokine IL-6. FACS analysis showed that only L13-Ia induced MHC II expression (Figure 3A). However, both strains induced IL-6 secretion, although the levels of secretion were significantly different (Figure 3B). Interestingly, IL-6 production was also induced by metabolites secreted by OLL2809 but not by L13-Ia (Figure 3B).

The analysis of oxidative stress markers indicated a significant decline in intracellular GSH (Figure 3C upper panel) and the lack of a detectable alteration in GSSG content (data not shown) in cells incubated with both strains of *L. gasseri*. However, a significant increase in GSHtot release resulted from MODE-K cell treatment with the L13-Ia strain compared to the control culture (Figure 3C lower panel).

### Modulation of IEC-iDC interaction

**Figure 4:**
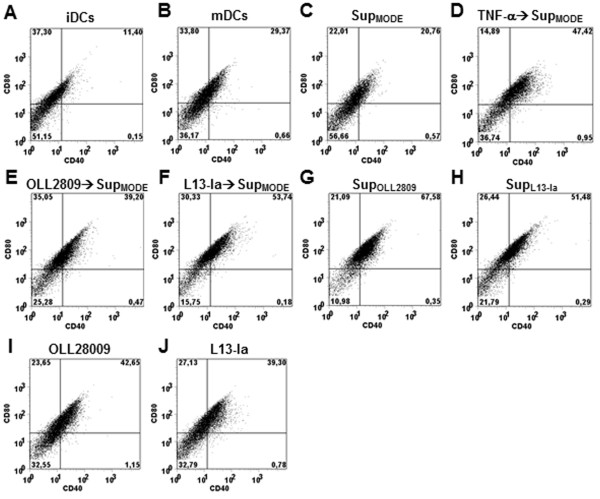
**Expression of co-stimulatory markers CD80 and CD40 on the surface of DCs conditioned with culture medium from MODE-K cells ±** ***L. gasseri *OLL2809/L13-Ia.** Before a 6-h LPS pulse, iDCs were challenged for 24 h with medium from: untreated MODE-K cell culture (Sup_MODE_, **C)**; MODE-K cells following TNF-α stimulation **(D)**; MODE-K cells following probiotic co-incubation **(E and F)**; irradiated OLL2809 or L13-Ia (24 h incubation; Sup_OLL2809_ and Sup_L13-Ia_, **G** and **H**). iDCs were also directly challenged for 24 h with irradiated bacteria **(I and J)**. iDCs **(A)** and untreated mDCs **(B)** were used as controls. DCs were stained for CD40 and CD80 and analyzed by FACS. Data were collected from ungated cells and are representative of three independent experiments.

**Figure 5:**
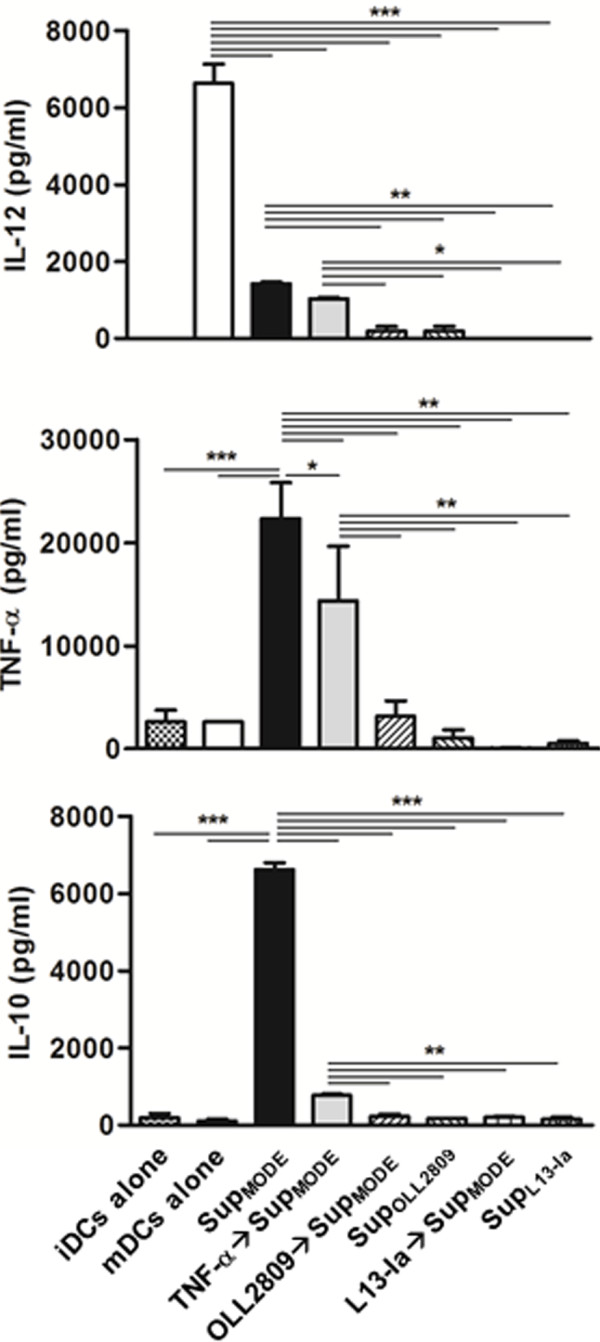
**Cytokine production by DCs conditioned with culture medium from MODE-K cells ±** ***L. gasseri *OLL2809/L13-Ia.** iDCs were challenged for 24 h with the same media described in Figure 4 and then LPS pulsed. IL-12, TNF-α and IL-10 levels were detected in the supernatants of DC cultures by sandwich-type ELISA; values are expressed in pg/ml; columns represent the mean ± SD and are representative of three independent experiments. Sup_MODE_ supernatant from 24 h culture of MODE-K cells; Sup_OLL2809_ and Sup_L13-Ia_, supernatants from irradiated bacteria incubated for 24 h in RPMI complete medium. *, *P* < 0.05; **, *P* < 0.01; ***, *P* < 0.001.

To evaluate the ability of IECs challenged by *L. gasseri* to instruct DCs, iDCs were incubated for 24 h with media conditioned by MODE-K monolayers in the presence or absence of *L. gasseri* strains. Following this treatment, iDCs were LPS pulsed and cultured for additional 24 h. As reported above, LPS increased expression of both CD80 and CD40 surface markers on DCs (Figure 4A-B). Pretreatment of DCs with supernatant from MODE-K monolayers (Sup_MODE_) down-regulated the expression of these markers (Figure 4C). However, down-regulation was completely reversed when MODE-K cells were stimulated with TNF-α (Figure 4D). Interestingly, bacteria-conditioned supernatants from MODE-K cells induced a further increase in the expression of the co-stimulatory markers (Figure 4E-F). The data reported in Figure 4G and H clearly showed that inductive effects also resulted from metabolites secreted into the medium by both bacterial strains (Sup_OLL2809_ and Sup_L13-Ia_). Direct challenge with bacteria was much less effective than challenge with the bacterial metabolites in inducing the expression of CD80 and CD40 on DCs following LPS stimulation (Figure 4I-J). We next examined the effects of conditioned media on the cytokine profile. Interestingly, Sup_MODE_ down-regulated IL-12 expression and markedly induced TNF-α and IL-10 in LPS-pulsed iDCs (Figure 5); this effect was dramatically reduced when MODE-K cells were treated with TNF-α. Notably, media from bacteria-conditioned MODE-K cell cultures completely suppressed the expression of all examined cytokines. A similar effect was reproduced when DCs were treated with Sup_OLL2809_ and Sup_L13-Ia_ (Figure 5). Baseline levels of IL-12, IL-10 and TNF-α in the various supernatants were undetectable, with the exception of TNF-α- > Sup_MODE_ where TNF-α levels were not significantly different from those found in the control (iDCs alone; data not shown). This indicated that added TNF-α (5 μg l^-1^) was mainly metabolized/degraded after 24 h in this sample. Direct incubation of iDCs with irradiated bacteria dramatically enhanced the secretion of all examined cytokines, after LPS pulse, at levels comparable to those reported in Figure 2 (data not shown).

### *L. gasseri* strains influence the antioxidant/cytoprotective defenses of DCs

**Figure 6:**
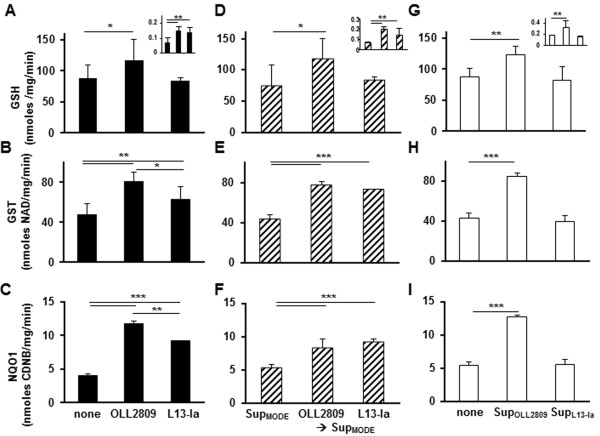
**Antioxidant/cytoprotective effects of *L. gasseri *OLL2809 or L13-Ia on LPS-pulsed DCs.** Intracellular and extracellular (upper inserts) thiol concentrations **(A, D, G)**, GST **(B, E, H)** and NQO1 activities **(C, F, I)** were measured in DCs challenged with irradiated strains (black bars), DCs exposed to conditioned media from MODE-K cells (Sup_MODE_, dashed bars) or DCs incubated with supernatant from irradiated bacteria (Sup_OLL2809_ and Sup_L13-Ia_, empty bars). LPS-pulsed DC culture was used as control. Extracellular thiols are expressed as nmoles/min. Intracellular GSH levels are expressed as nmoles/mg prot/min. GST and NQO1 activities were measured in cytoplasmic extracts and the obtained values, upon normalization to the protein content, were expressed as nmoles 1-chloro-2,4-dinitrobenzene (CDNB)/mg/min and nmoles NAD/mg/min, respectively; columns represent the mean ± SD and are representative of three independent experiments. *, *P* < 0.05 **, *P* < 0.01; ***, *P* < 0.001.

The effects on DC redox status and Nrf2-mediated cytoprotection elicited by the two *L. gasseri* strains were evaluated using LPS-pulsed DCs. In contrast to what was observed in IECs, a significant increase in intracellular GSH resulted from DC pre-exposure to OLL2809 compared to DCs treated with L13-Ia (Figure 6A), and GSH release in culture media was significantly increased by the presence of both *L. gasseri* strains (Figure 6A upper insert). Interestingly, significantly higher GST and NQO1 activities were found in DCs pre-exposed to both strains, although at different levels (OLL2809 > L13-Ia) (Figure 6B-C). When we examined the modulatory activities of bacteria-conditioned MODE-K cell culture on redox status and cytoprotective defenses, similar results were obtained, with the exception of a comparable increase of phase 2 enzyme activity operated by the two strains (Figure 6D-F). Importantly, Sup_MODE_ did not affect any of the examined antioxidant or cytoprotective parameters (Figure 6A-F). Finally, we examined the modulatory activities of Sup_OLL2809_ and Sup_L13-Ia_ on antioxidant/cytoprotective defenses in DCs. Interestingly, intracellular GSH content, GSH release in culture media and phase 2 enzyme activity in DCs were significantly upregulated by stimulation with Sup_OLL2809_ compared to stimulation with Sup_L13-Ia_ (Figure 6G-I). These treatments had no detectable influence on DC viability or intracellular GSSG concentration (data not shown).

## Discussion

In this study, we compared two probiotic strains of *L. gasseri*, OLL2809 and L13-Ia, and found that these strains had distinctive abilities to modulate *in vitro* mechanisms of innate immunity and antioxidant/detoxifying defenses.

OLL2809 was isolated from human feces [22]. The beneficial activity of this strain on mucosal inflammation has been previously shown in mice, where administration of OLL2809 was effective in reducing endometriotic lesions [30]. L13-Ia was isolated from raw whole bovine milk and was considered a potential probiotic strain [23] as it survived a selective *in vitro* digestion protocol. Another probiotic property of these strains has been confirmed in this study (Table 1).

The intestinal microbiota interacts with the local immune system promoting mechanisms of intestinal homeostasis [31]. Harnessing the contribution of probiotics to this physiological function has been proposed as a potential beneficial treatment for inflammatory bowel disease [32]. The activity of these probiotic organisms is thought to be mediated by the interaction of microbe-associated molecular patterns (MAMPs) with pattern recognition receptors (PRRs) on antigen-presenting cells. In particular, the immune response against lactobacilli is dictated by conserved MAMPs [33]. As a result of these interactions, some *L. gasseri* strains induce DCs to produce high levels of IL-10, IL-6, IL-12, and TNF-α [33]. In line with these data, herein we showed that direct exposure of *L. gasseri* strains to DCs resulted in strong cytokine responses with no deviation toward a specific phenotype. Notably, the reported pro-inflammatory phenotype of mDCs derived from this mouse strain [34] was abrogated after challenge with both *L. gasseri* strains as IL-10 was also induced. Nevertheless, all of these cytokines may contribute to innate immunity by inducing the proliferation and differentiation of natural killer cells *in vivo*[35].

In functional experiments, we set the bacteria: eukaryotic cell ratio to 30:1 on the basis of a study showing that this proportion was optimal to stimulate cells [36]. Using this protocol, a differential activity of the two *L. gasseri* strains was shown following bacteria challenge of mature DCs. This *in vitro* condition resembles the physiologic interaction occurring between bacteria and DC protrusions across the intestinal epithelium that reflects an active response to local commensal flora and bacterial products [29]. In our experiments, the percentage of CD11b^+^CD11c^+^ DCs and the expression of co-stimulatory markers (CD40 and CD80) were increased following maturation. Intestinal lamina propria (LP) DCs are classified into CD11c^hi^CD11b^hi^ and CD11c^hi^CD11b^lo^ DCs [37], which were found to be equivalent to CD103^+^CD8α^-^ and CD103^+^CD8α^+^ LPDCs subsets, respectively [38]. Interestingly, only OLL2809 sustained maturation of DCs in our experiments, leaving unchanged the percentage of CD11b^+^CD11c^+^ DCs and by increasing the expression of co-stimulatory markers.

We also examined the interaction of *L. gasseri* strains with intestinal epithelial cells (IECs). IECs, in addition to their metabolic functions, play a major role in the generation of innate immunity. To explore this function of IECs, we used a murine epithelial cell line (MODE-K) derived from the small intestine [24]. We found that the two *L. gasseri* strains differentially influenced MODE-K cells. In particular, OLL2809 was more effective than L13-Ia in stimulating IL-6 secretion without inducing surface expression of MHC class II molecules. However, L13-Ia induced the expression of MHC class II, a phenomenon that allows IECs to stimulate CD4^+^ T cells during inflammation or in response to infection. Moreover, only Sup_OLL2809_ induced IL-6 secretion in MODE-K cells, thus further highlighting the existence of distinctive responses elicited by these strains. The biological significance of the IL-6 increase remains controversial because this cytokine has both pro-and anti-inflammatory activities. Its receptor, IL-6R, is expressed on the surface of only a few cell types including hepatocytes and some leukocytes. The IL-6/IL-6R complex associates with gp130, which dimerizes and initiates intracellular signaling that triggers anti-inflammatory activities, such as inhibition of apoptosis and a parallel induction of proliferation in IECs [39]. However, IL-6 trans-signaling appears to mediate the pro-inflammatory activity of this cytokine, a process involving the binding of the soluble form of IL-6R to gp130 on cells that do not express IL-6R [39]. Our findings suggest that OLL2809 might contribute to gut immune homeostasis better than L13-Ia. Moreover, our results strengthen the concept that a probiotic activity can be induced not only from whole microorganisms and cell wall components but also from secreted metabolites. Stabilization of the enterocyte cytoskeleton was found to be mediated by a protease-sensitive metabolite secreted by the probiotic mixture VSL#3 [40]. More recently, exposure to probiotic-conditioned media was shown to attenuate the inflammatory responses induced in different enterocyte models [41].

In the intestinal lamina propria, DCs are classically immature DCs that, following antigen encounter, migrate into mesenteric lymph nodes where they are primed. The existence of IEC-DC crosstalk has been suggested by observations showing that IECs can drive differentiation of Treg cell-promoting DCs. This differentiation is mediated by IEC-secreted transforming growth factor-β and retinoic acid [42]. In agreement with these findings, we confirmed that medium conditioned by unstimulated MODE-K cells induced a regulatory phenotype in DCs, as shown by the reduced surface expression of co-stimulatory markers and, most importantly, reversal of the IL-12/IL-10 ratio. In the presence of a pro-inflammatory stimulus (i.e., treatment with TNF-α), this regulatory phenotype was abrogated, confirming that IEC-DC crosstalk is highly regulated. We further addressed this issue by evaluating the ability of *L. gasseri* strains to modulate the IEC-DC interaction. Our data indicate that both strains influenced IEC-DC crosstalk with distinct outcomes compared to those induced by Sup_MODE_. In particular, these strains markedly enhanced the expression of co-stimulatory markers and downregulated IL-12, TNF-α and IL-10 secretions by mDCs. In addition, similar alterations were induced by Sup_OLL2809_ and Sup_L13-Ia_, thus excluding a synergistic effect of IECs. However, our model does not allow us to further elucidate this probiotic activity because MODE-K cells do not form a confluent monolayer, instrumental to analyze the different roles played by paracellular and transcytosis pathways [42]. Taken together, our data suggest that the *L. gasseri* influence on IEC-DC crosstalk is dominant over IEC activity. Importantly, MODE-K cells and *L. gasseri* are able to produce different outcomes, regulatory mDCs and “low-responsive” mDCs, respectively.

Another beneficial effect on host immunity arising from the interaction between epithelia and commensal bacteria is the generation of reactive oxygen species that may activate the Nrf2 pathway and lead to improved antioxidant/detoxifying defenses [6]. The Nrf2-Keap1 complex serves as an intracellular oxidative stress sensor, and Nrf2 release, triggered by mild ROS production, activates the synthesis of a battery of cytoprotective/defensive proteins including GSH, GST and NQO1 that protect cells against oxidative stress and promote cell survival [5]. GSH plays a key role in the maintenance and regulation of the cell’s redox status. Our data showing opposing effects of bacterial strains on MODE-K cells’ and DCs’ intracellular GSH content are consistent with the reported pro-oxidant activity exhibited by probiotic strains [6] and with the improved ability of DCs to survive in an oxidant-rich environment [43]. Under normal conditions, intracellular GSH levels depend upon the rates of GSH synthesis/oxidation and on GSH export from cells, and the GSH/GSSG pair is widely used as an indicator of redox status. Data from this study on MODE-K cells, according to the literature, indicates that the lack of intracellular GSSG accumulation is associated with depletion and increased export of intracellular GSH [44]. In contrast, the increased intracellular GSH concentration accompanied by the increase in GSHtot export from the DCs without any measurable raise of intracellular GSSG concentration indicates the ability of DCs to respond to *L. gasseri*-induced oxidative stress by increasing GSH synthesis. These results, along with the results showing the improvement of GST and NQO1 activities in DCs directly exposed to *L. gasseri* strains or to conditioned supernatants from MODE-K cells, along with *in vivo* studies, further support the ability of bacterial strains to activate the Nrf-2 pathway [8,9].

## Conclusions

We have demonstrated *in vitro* differential immunomodulatory activities of two probiotic strains of *L. gasseri*, isolated from different sources. Our data also highlight the potential of secreted metabolites derived from these strains to influence IEC-DC crosstalk, Nrf2-mediated cytoprotection and consequently, gut immune homeostasis. Whether bacterial cell wall breakdown products or secreted molecules were responsible for this phenomenon is under investigation to aid in applications aimed at the amelioration of specific immunological conditions.

## Abbreviations

DCs: Bone marrow-derived dendritic cells; IECs: Intestinal epithelial cells; GSH: Glutathione; GSTs: Glutathione S-transferases; MAMPs: Microbial-associated molecular patterns; NQO1: NAD(P)H:quinone oxidoreductase; Nrf2: Nuclear erythroid 2-related factor 2; ROS: Reactive oxygen species; Supmode: Supernatant from MODE-K monolayers; SupOLL2809/L13-Ia: Supernatant from bacterial strains; γGCL: Gamma-glutamylcysteine ligase.

## Competing interests

The authors declare that they have no competing interests.

## Authors’ contributions

DL, PB, ST and MR conceived the study; MR and ST designed the study; DL, JM, PB and FN did the laboratory work; DL, JM, PB, FB, TS, ST and MR analysed the data; DL, PB, and MR wrote the manuscript; all authors read and approved the final manuscript.
